# Cardiovascular Abnormalities and Mental Health Difficulties Result in a Reduced Quality of Life in the Post-Acute COVID-19 Syndrome

**DOI:** 10.3390/brainsci11111456

**Published:** 2021-11-02

**Authors:** Catalina Giurgi-Oncu, Cristina Tudoran, Gheorghe Nicusor Pop, Cristina Bredicean, Silvius Alexandru Pescariu, Ana Giurgiuca, Mariana Tudoran

**Affiliations:** 1 Department VIII Neurosciences, Discipline of Psychiatry, “Victor Babes” University of Medicine and Pharmacy Timisoara, No. 2 E. Murgu Square, 300041 Timisoara, Romania; catalina.giurgi@umft.ro (C.G.-O.); bredicean.ana@umft.ro (C.B.); 2 “Pius Brinzeu” County Emergency Hospital, 300723 Timisoara, Romania; tudoran.mariana@umft.ro; 3 Department VII, Internal Medicine II, Discipline of Cardiology, “Victor Babes” University of Medicine and Pharmacy Timisoara, No. 2 E. Murgu Square, 300041 Timisoara, Romania; 4 Center of Molecular Research in Nephrology and Vascular Disease, Faculty of Medicine, “Victor Babes” University of Medicine and Pharmacy Timisoara, No. 2 E. Murgu Square, 300041 Timisoara, Romania; 5 Department VI, Discipline of Cardiology, “Victor Babes” University of Medicine and Pharmacy Timisoara, No. 2 E. Murgu Square, 300041 Timisoara, Romania; pop.nicusor@umft.ro (G.N.P.); pescariu.alexandru@umft.ro (S.A.P.); 6 “Dr. Victor Popescu” Military Emergency Clinical Hospital, 300080 Timisoara, Romania; 7 Department of Clinical Neuroscience, “Carol Davila” University of Medicine and Pharmacy, 050474 Bucharest, Romania; ana.giurgiuca@umfcd.ro; 8 “Prof. Dr. Al. Obregia” Psychiatric Clinical Hospital, 041914 Bucharest, Romania

**Keywords:** post-acute COVID-19, mental illness, anxiety, depression, cardiovascular complications, quality of life

## Abstract

(1) Background: Post-acute COVID-19 syndrome, characterized by persisting symptoms up to 12 weeks after the acute illness, impairs numerous people’s physical and mental health. (2) Methods: 64 inpatients and 79 outpatients, aged under 55 years, with post-acute COVID-19, were evaluated by a transthoracic echocardiography (TTE), mental health examination, Quality of Life (QoL) questionnaire, post-COVID-19 functional status scale (PCFS) and Hospital Anxiety and Depression Scale (HADS). (3) Results: all inpatients had mild/moderate pulmonary injury during acute COVID-19, in contrast to 37.97% of outpatients. Inpatients who reported an average of 5 persisting symptoms, had, predominantly, level 3 PCFS and a median QoL of 62, compared to outpatients, who reported an average of 3 symptoms, level 1 PCFS and a median QoL score of 70. Increased pulmonary artery pressure was detected in 28.11% of inpatients, compared to 17.72% of outpatients, while diastolic dysfunction was diagnosed in 28.12% of inpatients, in comparison with 20.25% of outpatients (*p* = 0.02). Abnormal systolic function was assessed in 9.37% of inpatients, and 7.58% of outpatients. According to the HADS depression subscale, 46.87% of inpatients and 27.84% of outpatients had clinical depression. Concomitantly, anxiety was detected in 34.37% of inpatients and 40.5% of outpatients (4) Conclusions: cardiovascular and mental health difficulties were frequently detected in patients with post-acute symptoms of COVID-19, which correlated with the number and intensity of persisting symptoms and reduced QoL scores.

## 1. Introduction

In recent months, since the start of the coronavirus (COVID-19) pandemic, it has become increasingly evident that there are far more health sequelae than previously suggested, even after an apparent recovery from the acute phase of the infection with the severe acute respiratory syndrome coronavirus 2 (SARS-CoV-2) [1]. In the aftermath of this unprecedented global health crisis, there are increasing numbers of patients who report, even at 3 months after the acute phase of illness, persisting distress in association with nonspecific, wide-ranging and, sometimes, even debilitating, residual symptoms, such as fatigue, dyspnea/persistent oxygen requirement, chest pain, post-viral chronic malaise, headaches, neurocognitive (brain fog), and mental health difficulties, such as anxiety, depression, disturbed/nonrestorative sleep, or psychotic episodes [2,3].

This clinical presentation is not dissimilar to that described by survivors of the SARS epidemic from 2003 [2,4]. Essentially, COVID-19 patients may be divided into those who may suffer severe pulmonary, thromboembolic, cardiovascular (CV), and neurological complications, and those with a non-specific, milder, clinical picture [3]. The most common complications are pulmonary fibrosis and CV abnormalities, ranging from severe, such as pulmonary thromboembolism, frequently associated with right ventricular dysfunction (RVD), arrhythmias, myocardial ischemia, acute myocarditis with heart failure (HF), to mild dysfunctions, such as sinus tachycardia or hypotension, which might explain the persistence of symptoms during recovery. However, neurological and mental disorders have also been frequently described long after the improvement from the acute phase of COVID-19 and may contribute to a persistently impaired health status [5,6,7,8].

Most of these subacute cardiovascular complications may be explained by delayed inflammatory and immunologic responses, involving an increased release of pro-inflammatory cytokines, such as interleukin-1α, tumor necrosis factor-α, interleukin-6, alongside C-reactive protein, a non-specific inflammatory biomarker. Notably, these delayed inflammatory and immunologic responses have also been identified as part of the COVID-19 illness, as well as in patients with major depressive disorder.

Provided the likely common underlying substrates subserving both CV disease and mental illnesses, such as major depressive disorder, including, but not limited to the production of stress hormones, may have a role in the prolonged effects of the post-acute COVID-19 syndrome. More specifically, the use of high corticosteroid doses in the treatment of acute COVID-19 infection may suggest that infection with COVID-19 activates the hypothalamic–pituitary–adrenal (HPA) axis and negatively affects the central serotonin system, resulting in mood dysregulation post-acute infection.

As an inevitable consequence of the constant state of global health crisis, and the permanent state of tension and fear in the face of an uncertain future, coupled with social/physical isolation recommendations, which may be considered abnormal for the so-called “social” human being, the mental health of the general population will be affected. Preliminary evidence suggests that symptoms of anxiety and depression (16–28%) and self-reported stress (8%) are common psychological reactions to the COVID-19 pandemic and may be complicated by a disturbed sleep pattern and a reduced overall quality of life [9]. The 60-day, post-acute COVID-19 American study [10] analyzed medical records and telephone follow-up evaluations of 1250 discharged patients and concluded that 6.7% of patients died and 15% required re-admission, while 32.6% were struggling with persistent symptoms, such as effort dyspnea, cough, anosmia, and ageusia.

In the absence of specific classification and diagnostic guidelines, according to some authors, the current definition of post-acute COVID-19 suggests a condition extending beyond 3 weeks from the onset of symptoms, while chronic COVID-19 extends beyond 12 weeks [2,10,11]. As several authors have now highlighted, there are increasing numbers of patients diagnosed with delayed recovery from COVID-19, leading to the current focus on its post-acute episode, which appears to be a multisystem disease occurring in the aftermath of a, not necessarily, severe pulmonary injury during the acute illness [2,3,10].

Recent theories on the clinical management of this post-acute illness suggest the need for a holistic perspective [12], considering the frequency and relative importance of the consequences, priorities, and specificity of interventions required for each impairment. The aim of recent and ongoing studies is also to improve the early identification of at-risk groups for developing post-acute COVID-19 [13].

This study aims to highlight the frequent association between the severity of the acute illness, expressed by the degree of the initial pulmonary injury and the inflammatory response, with the consecutive amplitude of CV and mental health impairments, as well as their impact on the quality of life in subjects who were hospitalized or treated as outpatients for a mild/moderate SARS-CoV-2 pulmonary infection and are currently suffering from post-acute COVID-19. Another aim was to analyze the influence of hospital admissions on the subsequent mental health and wellbeing of in- and outpatients during the acute phase of COVID-19, which will be essential in informing integrated healthcare and recovery guidelines and policymakers.

## 2. Materials and Methods

### 2.1. Study Population

This study initially screened 325 individuals who suffered from COVID-19 less than 3 months before attending the specialized outpatient clinic of a large clinical and emergency hospital in the west of Romania between January and March 2021 for concerns in the form of persisting symptoms (such as fatigue, dyspnea, heart palpitations, chest pain, reduced exercise capacity, headaches, neurocognitive difficulties, anxiety, depression, and disturbed sleep), all reporting that they have not yet returned to their basal health status. We selected 214 patients younger than 55 years old without a history of significant chronic diseases (especially CV pathology or mental illnesses) of all the service-users who had been hospitalized or treated as outpatients for COVID-19 during the second outbreak of COVID-19 (end of September 2020–beginning of January 2021), to be included in this study. Of this subgroup, only 157, with a mild/moderate SARS-CoV-2 pulmonary infection, were considered suitable for our study. After being informed about the purpose of this future research, 152 of them agreed to take part and signed informed consent forms. Of these patients, a further 9 subjects were excluded, either due to insufficient data referring to the evaluation of the acute infection or due to significant preexisting chronic health issues discovered during the cardiologic or mental health evaluation performed in the study.

**Figure 1:**
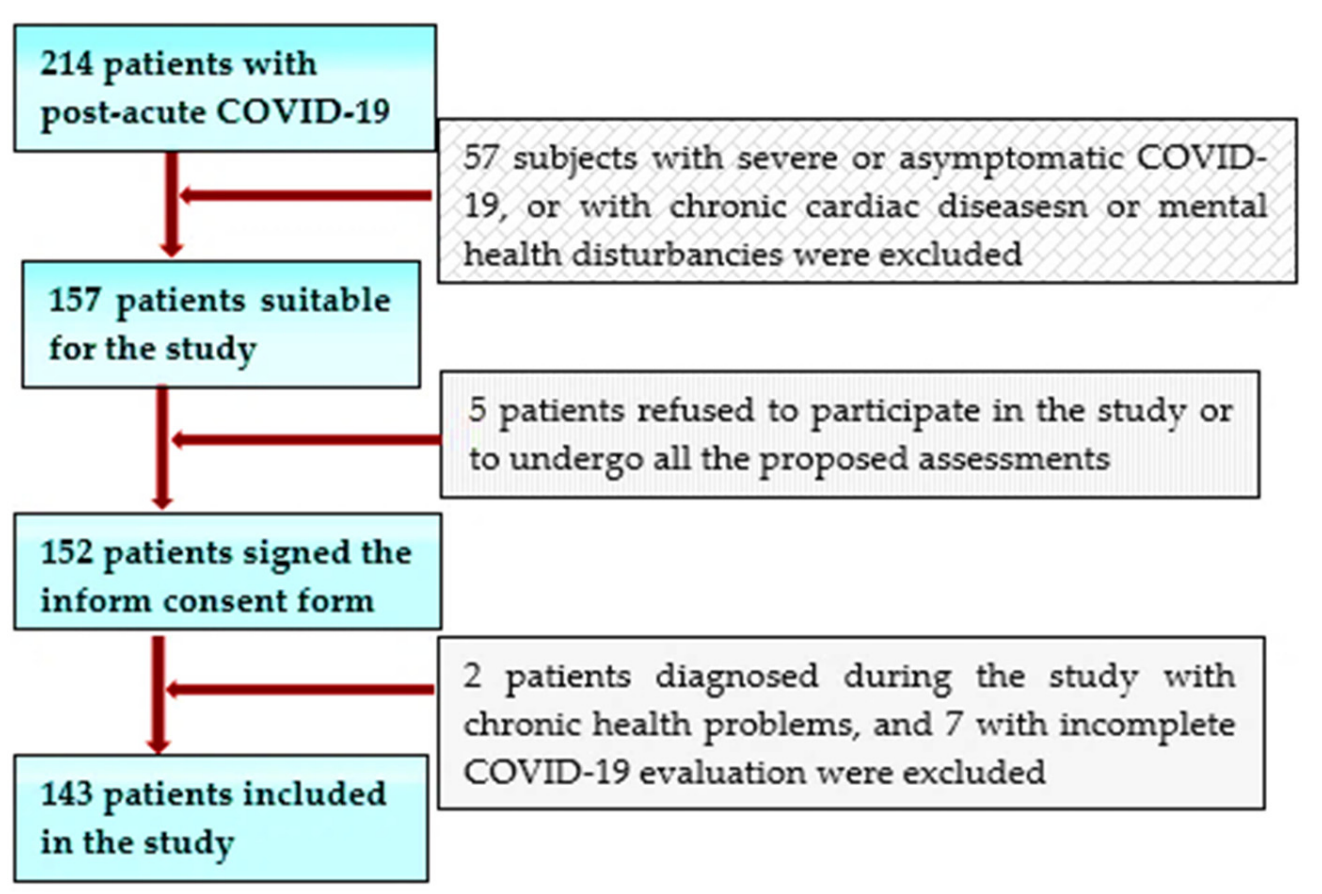
Timeline for the selection of the study population.

The inclusion criteria were as follows: patients aged over 18 years; the capacity to sign an informed consent; a recent SARS-CoV-2 infection certified by a positive result of real-time reverse transcriptase–polymerase chain reaction (RT–PCR) assay of nasal and pharyngeal swabs within 3 and 12 weeks before inclusion in the study; and subjects who were hospitalized for COVID-19 or had, at least, an initial clinical, biological, or thoracic computer-tomographic (TCT) assessment during the acute phase of the disease, and who agreed to have a cardiological assessment by transthoracic echocardiography (TTE) to attend a mental health examination, as well as to complete the Quality of Life (QoL) EQ-5d-5L scale and the Hospital Anxiety and Depression Scale (HADS) (Figure 1).

Exclusion criteria consisted of people aged over 55 years (with a higher likelihood to suffer from chronic diseases or to have age-related abnormalities on the TTE); patients already diagnosed with CV diseases or suffering from a chronic mental illness; individuals without an initial COVID-19 assessment; those who had asymptomatic or severe forms of this disease, with respiratory insufficiency requiring ventilation support or hospitalization in critical care units; and patients who suffered recent stressful life events (significant loss) in the context of the pandemic (Figure 1).

This study was approved by the Ethics Committee of the “Pius Brinzeu” County Emergency Hospital, Timisoara, Romania (No. 206/7 September 2020).

Subsequently, the demographic data, medical history, and results from the discharge summary or the outpatient clinical evaluation files were collected for all of study participants. Additionally, all individuals underwent comprehensive clinical examinations, electrocardiograms, and transthoracic echocardiography (TTE) assessments. Patients were classified according to the Post-COVID-19 Functional Status (PCFS) scale, and, afterward, all of them were asked to complete the QL-5D-5L quality of life questionnaire and were further referred to a mental health professional for a complete mental health evaluation.

### 2.2. Methods

The initial COVID-19 assessment was performed, as per our inclusion criteria, either during the hospitalization or on an outpatient basis, and included a clinical examination, thorax computer tomography (TCT) with the evaluation of pulmonary injuries by determining the extent of parenchymal involvement as mild (<25%), moderate (25–50%), or severe (>50%) or by the degree of lung aeration [14], daily O_2_ assessment by pulse-oximetry and laboratory blood analysis, such as complete blood count (CBC), C-reactive protein (CRP), ferritin levels, glycaemia, aspartate aminotransferase (AST), and alanine aminotransferase (ALT). A mild form of COVID-19 meant a pulmonary injury of under 0–25% on TCT and mild symptoms (cough, fever, changes in smell or taste) without dyspnea, while a moderate one was characterized by an O_2_ saturation of over 94% and a pulmonary injury between 25–50%.

The cardiological evaluation comprised the personal medical history, assessing all risk factors, clinical examination, electrocardiogram, and transthoracic echocardiography (TTE), performed according to guideline recommendations [15].

TTE examinations included a complete assessment of the cardiac morphology and function, followed by the evaluation of the left and right ventricular performance, by measuring the following parameters: (1) The left ventricular (LV) systolic and diastolic performance, which was evaluated in 2D mode, from apical 2-, 3-, and 4-chamber view, by determining the LV ejection fraction (LVEF), based on the modified Simpson method (with values below 50% considered pathological), and the lateral mitral annular plane systolic excursion (a MAPSE below 10 mm was considered abnormal). (2) The diastolic dysfunction (DD) was assessed from an apical 4-chamber view, in pulsed Doppler, at the level of the mitral valve annulus, by registering the peak early diastolic velocity (E), the late diastolic velocity (A), and an E/A ratio, which was subsequently calculated. Next, we employed a Tissue Doppler imaging (TDI) to record the early (e’) and the late diastolic velocity (a’), at the level of the septal and lateral mitral annulus and calculated an average E/e’ ratio. An E/A ratio ≤ 0.8 and E ˂ 50 cm/s defined a type 1 DD, while an E/A ratio of over 2 indicated a type 3 DD. In the case of an E/A ratio ≤ 0.8, with an E of over 50 cm/s, or if the E/A was between 0.8 and 2, a type 2 DD was presumed, and certification was required, by fulfilling at least 2 of the following criteria: an average E/e’ > 14, a left atrial volume index (LAVI) of over 34 mL/m^2^ and a tricuspid regurgitation velocity (TRV) of over 2.8 m/s. In those cases, where only 1 of these 3 criteria was fulfilled, we diagnosed a type 1 DD [16]. (3) The right ventricular (RV) function was assessed in an apical 4-chamber view by determining the fractional area change (FAC—values under 35% were considered as pathological), and the tricuspid annular plane systolic excursion (TAPSE), measured in M-mode, at the level of the lateral tricuspid valve annulus (values under 17 mm defined an RVD). (4) The estimated systolic pressure in the pulmonary artery (sPAP) was assessed from the apical window at the level of the tricuspid valves, in continuous-wave Doppler, by measuring the peak tricuspid regurgitation velocity (TRV) and estimating the right atrial pressure, based on the inferior vena cava diameter, and its respiratory variations. In this study, we considered that sPAP values of ≥35 mmHg at rest indicated pulmonary hypertension (PH) with severities ranging from mild (35–44 mmHg) to moderate (45–60 mmHg) to severe (>60 mmHg) [17,18].

The Post-COVID-19 Functional Status (PCFS) scale is an evaluation method developed to assess the recovery status after COVID-19, where 0 means “no limitations and symptoms”; 1—“negligible limitations of usual activities with persistent symptoms”, 2—“slight limitations with significant symptoms”, 3—“moderate limitations and not able to perform all usual activities due to symptoms, but still able to take care of him/herself without assistance” and 4—“severe limitation due to symptoms and requiring assistance to take care of themselves” [19]. All patients were classified according to this system in order to identify the severity of their functional limitations.

The EQ-5D-5L QoL questionnaire is a self-evaluation instrument which allows a quantitative assessment of 5 current health-related domains, including mobility, self-care, ability to perform usual activities, pain/discomfort, and anxiety/depression. Scores are indicative of the patient’s status, based on the EQ visual analog scale (VAS), with the endpoints labelled as “The best health you can imagine” and “The worst health you can imagine” and with 5 potential severity levels (no problems, slight problems, moderate problems, severe problems, and extreme problems) [20].

For patients who reported associated psychological distress, there was a referral to the mental health team, who completed an evaluation. Clinical details were corroborated with the results obtained using the Hospital Anxiety and Depression Scale (HADS). For the validity of results, we ensured that we only included patients without a prior mental health history and without any significant recent stressful life events in the context of the pandemic (such as significant personal or financial loss) during the baseline assessment.

The Hospital Anxiety and Depression Scale (HADS), developed by Zigmond and Snaith [21], is a self-assessment scale, which consists of 14 questions divided into 2 7-item subscales, relating to personal concerns regarding the intensity of depression (HADS-D), and anxiety (HADS-A), respectively. When devising the scale, the authors deemed the mixture of anxiety and depression items as necessary, to be able to reflect the frequent intersection of the two, often indistinguishable, mental health conditions. Potential scores for each item are based on a 4-point Likert scale, with possible answers extending from 0 = never to 3 = always. Scores ranging from 8–10 indicate the presence of mild depression/anxiety, scores between 11–14 suggest a moderate intensity of clinical symptoms, while scores between 15 to 21 indicate a severe intensity of symptoms.

Statistical methods: The Statistical Package for the Social Sciences v.25 (SPSS, Chicago, IL, USA) was employed to perform data analysis. Continuous variables were presented as mean and standard deviation (SD) or median and interquartile range (IQR), and categorical variables as frequency and percentages. Because the results of the normality test (Shapiro–Wilk) showed a non-Gaussian distribution, we continued the analysis by using nonparametric tests. We employed the Mann–Whitney U test to compare our groups of patients, and the Chi-square test or Fisher’s exact test to evaluate the significance of differences in the proportions of nominal variables. We employed Spearman’s correlation test to assess the potential connection between number of symptoms and QoL scores with TTE parameters, laboratory results, HADS D, and HADS A scores. We considered that *p* values of under 0.05 would indicate statistically significant differences.

## 3. Results

This study included a total of 143 patients (65 men and 78 women) aged between 18 and 55 years (mean age 44.06 ± 9.12 years), who suffered from a mild/moderate COVID-19 infection within 4 to 12 weeks before this investigation. We ensured that none of them had a history of significant CV diseases or a previous chronic mental illness. Based on the persistence of symptoms, all patients were diagnosed with post-acute COVID-19. Their clinical and laboratory data, their PCFS levels, QoL, HADS-D, and HADS-A scores are presented in Table 1.

In terms of the required healthcare in the acute phase of illness, subjects were divided into two subgroups: outpatients and inpatients. The first subset comprised 64 hospitalized subjects (31 men and 34 women), aged between 29 and 55 years, with a median age of 46 [41–52] years old, hospitalized for COVID-19 pneumonia (27 of them with a mild form of illness and 37 with a moderate form of illness), with a TCT-assessed pulmonary injury of between 10% and 37%. There were no statistically significant differences regarding age and gender. The duration of hospitalization was between 8 and 17 days, with a median inpatient stay of 14 (10–15) days. All subjects who reported between 3 and 9 persisting symptoms, with an average of 5 (4–6) symptoms, were classified as having PCFS levels between 1 (3 patients), 2 (33 cases), and 3 (28 subjects), and reported QoL scores ranging from 31 to 85, with a median of 62 (48–74.75).

The echocardiographic assessment determined a reduced LVEF (under 50%) and a reduced MAPSE in 6 subjects (9.37%); thus, the median of LVEF in the whole subset was of 55 (52–61). Eighteen patients (28.12%) were diagnosed with DD based on elevated values of the E/e′ ratio. Elevated sPAP values were calculated in 18 patients (28.11) with levels between 40 and 48 mmHg and a median of 34 [29.25–42], while 22 subjects (34.37%) showed a reduced FAC.

After analyzing the results from the HADS evaluation, we identified the presence of depression in 30 subjects (46.87%), with HADS-D scores between 14 and 21,22 (34.37%) with associated anxiety, and another 8 with borderline anxiety scores. A further 30 patients (46.87%) had borderline depression scores of over 7, half of which also scored in line with comorbid borderline anxiety; only four patients (6.25%) had standard scores.

The second subset included 79 outpatients (34 men and 45 women), aged between 18 and 55 years, with a median age of 42 (36–52) years, the majority of which suffered from a mild form of COVID-19, with only 1 patient having been diagnosed with a moderate form. Thirty of them (37.97%) had pulmonary injuries diagnosed by TCT. Patients reported between two and four persisting symptoms, with an average of three (2–4). Fifty-five subjects were classified as level 1, another twenty as level 2, and the remaining four as level 3, with respect to the PCFS levels. QoL scores ranged between 45 and 85, with a median of 66 (60–75).

On the TTE evaluation, we determined a reduced LVEF in 6 subjects (7.59%), with a median for the whole subset of 56 (54–65); a DD was diagnosed in 16 patients (20.25%), while a further 14 (17.72%) had elevated values of sPAP of between 39 and 46 mmHg, and a median of 30 (24–34); a further 16 patients had slightly reduced FAC levels, with a median of 36.43 (35.47–37.32).

The results of the HADS evaluation demonstrated elevated HADS-D levels in 22 subjects (27.84%), who also reported associated anxiety. In a further 10 participants (12.65%), we determined increased values of the HADS-A scores, 8 of which had associated borderline depression. In the remaining 47 subjects, 26 had borderline levels of anxiety, 4 of which had associated borderline depression, and another 4 scored significantly only for borderline depression. There were only 17 subjects (21.51%) who did not report symptoms of anxiety or depression.

The statistical analysis of these results did not show any significant differences regarding age and gender distribution between the two groups, while BMI was significantly higher in the first subset. When referring to the initial COVID-19 assessment, the hospitalized patient group had significantly more severe pulmonary injuries, higher levels of CRP, lower oxygen saturation, and more accelerated heart rates. Generally, they were examined sooner after the acute illness, reported significantly more symptoms, had higher PCFS scores and worse TTE parameters. Although hospitalized patients reported higher levels of anxiety, the difference was not statistically significant; by contrast, in terms of the HADS depression scale, the first category of patients (hospitalized) had significantly higher levels of depression and reported lower VAS scores.

Further statistical analysis by Spearman’s correlation indicated strong statistically significant correlations between the number of persisting symptoms and the number of weeks since the COVID-19 diagnosis, the severity of the pulmonary injury, the CRP levels during the acute infection, and the number of hospitalization days (*p* ˂ 0.001) (Table 2). Similar correlations with the PCFS scale and the HADS-D levels (*p* ˂ 0.001) were identified. The correlations with TTE parameters, such as PAPs, FAC, LVEF, E/e’ and HADS-A levels, were moderate, but also statistically significant (*p* ˂ 0.001) (Table 2). Other significant correlations were evidenced between QoL scores, the number of weeks since diagnosis, severity of the pulmonary injury, and CRP levels during the acute illness, as well as the PCFS scale (*p* ˂ 0.001). Similar correlations with the HADS-D and -A levels (*p* ˂ 0.001) were observed. We also found significant correlations with TTE parameters, such as PAPs, FAC, LVEF, and E/e’ (*p* ˂ 0.001), Table 3.

**Figure 2:**
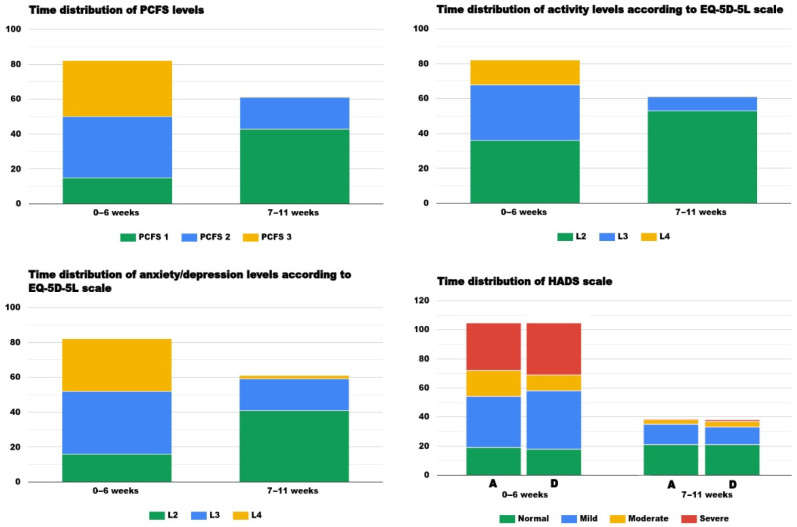
Time distribution of the results of the PCFS, EQ-5D-5L, and HADS scales evidencing that patients assessed in the first 6 weeks after the COVID-19 diagnosis had worse levels of PCFS, a lower level of activity, and reported more severe symptoms of anxiety and depression in comparison to those evaluated after 7 to 11 weeks of recovery.

When the results of our assessments were analyzed by taking into account the time elapsed from the acute illness until the moment of the evaluations, we noticed that patients who were examined sooner than 6 weeks since the SARS-CoV-2 infection had significantly lower functioning scores, more severely impaired activity levels, and more pain/discomfort. They also reported increased anxiety and/or depression levels, with worse VAS scores compared to the subjects assessed from weeks 6 to 11 (Figure 2, Table 3). This result may be, in part, explained by the fact that hospitalized patients, who generally had a more severe form of the disease, were more symptomatic and more likely to come for a medical examination earlier.

## 4. Discussion

Evidence from recent medical literature suggested that there are growing numbers of patients diagnosed with a delayed recovery from COVID-19, leading to the current focus on its post-acute episode, which appears to be a multisystem disease occurring as a repercussion of a, not necessarily severe, form of the acute illness [3]. While still lacking specific classification and diagnostic guidelines, some authors state that the accepted definition of post-acute COVID-19 proposes a condition extending beyond 3 weeks from the onset of symptoms, while long COVID-19 is defined as extending beyond 12 weeks [11].

According to the British COVID-19 study [1], roughly 10% of patients who have tested positive for SARS-CoV-2 remain unwell beyond 3 weeks, while a smaller but significant proportion continue to suffer for months [22]. A recent American study posited that 35% of people had not returned to their previous level of health 3 weeks after a positive test [23]. Other observational studies [22,24] give an even grimmer forecast, stating that almost 50% of infected individuals had difficulties resuming their normal activity levels, which might be partially explained by research mainly focused on populations who required hospital admissions or healthcare in specialist clinics.

Furthermore, concerns have been raised about the mental health reverberations since the onset of the COVID-19 pandemic, alongside the focus on the vital somatic implications of the illness [25]. According to an observational study of a cohort of 60,000 patients with COVID-19, within 6 months after the illness, 1 out of every 3 patients had been diagnosed with a mental or neurological disorder. Researchers also looked at the medical records of 230,000 American survivors of COVID-19 and found that 34% of them experienced neuropsychological difficulties. Of these, 13% had no recorded personal history of a mental illness. The study found that the most common problems were anxiety and mood disorders, affecting 17% and 14% of people, respectively. A small percentage of the cohort were diagnosed with new-onset psychosis or neurological disorders, such as stroke and dementia, with the caveat that these diagnoses were made only in the cases of people with severe forms of the infection that required admission into an intensive care unit (ICU) [5,26]. The post-acute “brain fog” neurocognitive difficulties of people that required ICU stays might be explained by deconditioning or PTSD [27], alongside dysautonomia that could be a factor in similar concerns experienced by people who suffered mild forms of the illness [28].

However, a recent French study that evaluated 150 people at 60 days after disease onset found that two-thirds of patients still experienced persistent dysfunctions, while a third of them reported worse clinical presentations than at onset [13].

In terms of the potential contextual triggers for the anxiety and mood disorders, they might be understood as a “perfect storm” that formed as an association of long-lasting personal health and safety concerns and concerns about the wellbeing of loved ones, coupled with the unusually long periods of quasi-institutionalized, imposed social/physical distancing, which is uncanny for the “social” human being. However, questions remain unanswered about the post-viral neurological and neuropsychiatric consequences of COVID-19 [29,30].

This study intended to investigate the cardiovascular sequelae of COVID-19 and potential new-onset mental health symptoms and alterations of the individual QoL as a direct consequence of the illness. Our study inclusion criteria meant that we focused solely on people without a personal history of CV disease or chronic mental illness, in an effort to not confound the results. Based on the TTE assessment of our patient group, we frequently evidenced various cardiac abnormalities, with a predominance of PH and DD. An LV systolic dysfunction was only diagnosed in 8.5% of patients, which is comparable to other literature data [31].

The functional status of patients was appreciated based on the PCFS scale and the QL-5D-5L scale. Although according to both methods, none of our patients reported restrictions of their mobility and self-care abilities, all of them claimed various degrees of limitation in performing their usual activities due to persisting symptoms/discomfort and different degrees of anxiety/depression. This aspect was highlighted by the HADS-D and A subscales, with an increased prevalence of depression and anxiety in over a third of our study population. It must be mentioned that patients who were hospitalized for COVID-19 had higher depression and anxiety scores than those treated on an outpatient basis. However, we must consider that the latter group had a less severe form of the illness and may have not been exposed to highly upsetting situations inherent during hospitalization in dedicated COVID-19 wards. Although these three assessment scales may have certain superimposing items, together they offer a superior evaluation.

Another notable aspect was that the results of our assessments were worse in subjects evaluated sooner after the acute infection and tended to decrease in line with the weeks elapsed since.

As it appears that ever-increasing numbers of people report particularly challenging residual symptoms, both at the somatic level as well as when considering the toll on their mental health weeks after the acute phase was resolved, overall, these findings suggest that beyond the critical contribution of residual multisystem alterations, other factors are contributing to an ongoing impaired physical and mental status. This may be due to the direct effects of the infection and/or medication, but, quite possibly, also due to the unusual strict isolation measures, involving a persistent lack of direct contact with loved ones due to the banning of visitors. Patients describe feelings of abandonment as a direct consequence of the lack of visitors and the lack of direct medical professional–patient contact, which resulted in an inferior healthcare professional–patient alliance from the viewpoint of a previous abundance of contact throughout the inpatient stay, not to forget the impact of their suffering on the mental health of the medical and non-medical staff responsible for their care [32,33].

Data from a newly published systematic review that looked at the electronic health records of over 62,000 with a previous COVID-19 diagnosis indicated that the psychiatric consequences were broad and heterogeneous, with a higher incidence for all major anxiety disorder categories than for depression and other mood disorders. The authors assert that it remains unclear whether post-COVID-19 anxiety will have a particular post-traumatic stress disorder-like presentation, and circadian rhythm disturbances have also been prominently diagnosed [34]. However, cohort studies of patients with COVID-19 with adequate control groups and follow-ups are urgently needed to quantify the incidence of all psychiatric sequelae and relative risks after infection [35].

Current research efforts have vital significance when informing clinicians and policymakers in their joint work of outlining specific classifications and guidelines for the interdisciplinary approach towards a timely diagnosis and adequate management of the acute and rehabilitation phase of COVID-19. This complex pathology requires a holistic acute treatment and aftercare that would be better able to successfully negotiate the somatic sequelae while also addressing the mental health difficulties and help restore the patient’s health and overall quality of life [26].

The most important limitation of our study is the small number of patients, which was mainly a direct consequence of the challenge to identify patients with post-acute COVID-19 syndrome but without associated chronic diseases who were also willing to participate in our study. We should also highlight the important limitation of not having a control group (e.g., general inpatient vs. outpatient), as this would have also provided information on whether the dysregulation in mood was due to being an inpatient vs. outpatient rather than a consequence of persisting symptoms of post-acute COVID-19 infection. Another limitation is that we did not analyze our results concerning the severity of the disease and that we were not able to follow the evolution of our patients over time.

## 5. Conclusions

The post-acute COVID-19 syndrome represents an intricate multisystem pathology frequently associated with physical health sequalae alongside impaired mental health, with a significant impact on people’s wellbeing and quality of life. Fortunately, the number of symptoms and their intensity seems to decrease in parallel with the number of weeks elapsed since the acute phase of the illness.

## Figures and Tables

**Table 1:** Characteristics of study participants.

Characteristics	Hospitalized Patients = 64 (44.75%)	Outpatients = 79 (55.24%)	*p*
Age	46 [41–52]	42 [36–52]	0.106 ^a^
Gender: -men -women	31 (48.43%) 33 (51.56%)	34 (43.03%) 45 (56.96%)	0.519 ^b^
BMI	28.23 [24.67–34.5]	26.17 [22.56–29.4]	0.009 ^a^
Heart rate	75 [70–75]	70 [70–75]	0.002 ^a^*
Weeks since diagnosis	5 [5–6.75]	7 [6–8]	<0.001 ^a^*
Results of the first COVID-19 assessment			
Pulmonary injury on TCT	25 [10–30] (*n* = 64, 100%)	0 [0–5] (*n* = 30, 37.97%)	<0.001 ^a^*
Severity of COVID-19 –moderate –mild	37 (57.81%) 27 (42.18%)	1 (1.26%) 78 (98.55%)	<0.001 ^a*^
O_2_ saturation	97 [95–98]	97 [97–98]	0.001 ^a^*
CRP	36.54 [21.6–44.13]	24.57 [15.75–32.56]	<0.001 ^a^*
Days of hospitalization	14 [10–15]	-	-
Number of persisting symptoms	5 [4–6]	3 [2–4]	<0.001 ^a^*
PCFS scale	2 [2–3]	1 [1–2]	<0.001 ^a^*
Quality of life-VAS score	62 [48–74.75]	66 [60–75]	0.033 ^a^
HADS Depression	10 [10–19]	8 [6–11]	<0.001 ^a^*
HADS Anxiety	10 [7–16]	9 [7–13]	0.469 ^a^
Results of TTE assessment			
LVEF	55 [52–61]	56 [54–65]	0.256 ^a^
MAPSE	13 [11.25–13.75]	13 [12–15]	0.007 ^a^
E/A	1.23 [0.93–1.34]	1.35 [1.27–1.42]	<0.001 ^a^*
E/e′	11.61 [10.58–14.56]	11.05 [10.28–11.94]	0.002 ^a^*
sPAP	34 [29.25–42]	30 [24–34]	0.001 ^a^*
FAC	35.39 [33.69–36.1]	36.43 [35.47–37.32]	<0.001 ^a^*
TAPSE	19 [17.25–20]	20 [19–22]	<0.001 ^a^*

Legend: BMI—body mass index; TCT—thorax computer-tomography; CRP—C reactive protein; PCFS—post-COVID functional status; VAS—visual analog scale; HADS-D and HADS-A—Hospital Anxiety and Depression subscales for Depression and Anxiety; LVEF—left ventricular ejection fraction; MAPSE—mitral annular plane systolic excursion; sPAP—systolic pressure in the pulmonary artery; FAC—fractional aria change; TAPSE—tricuspid annular plane systolic excursion; E/A—peak mitral inflow early (E) to late (A) diastolic velocities in pulsed Doppler; E/e′—early mitral inflow diastolic velocity E to average e′ velocity (E/e′) in pulsed tissue Doppler;: []—interquartile range, () relative frequency; ^a^ Mann–Whitney U test; ^b^ Chi-square test/Fisher-exact test; *p* value ˂ 0.05 was considered statistically significant; * a significance threshold value of 0.05 was reached.

**Table 2:** Correlations between the number of persisting symptoms and quality of life scores with characteristics of the acute illness, echocardiographic and mental health parameters.

Parameter	Number of Symptoms		QoL VAS Score	
	*r* [95% CI]	*p*	*r* [95% CI]	*p*
Pulmonary injury	*r* = 0.840 [0.785; 0.888]	<0.001 *	*r* = −0.563 [−0.671; −0.420]	<0.001 *
CRP levels	*r* = 0.729 [0.623; 0.807]	<0.001 *	*r* = −0.719 [−0.804; −0.611]	<0.001 *
Hospitalization days	*r* = 0.735 [0.662; 0.799]	<0.001 *	*r* = −0.329 [−0.473; −0.160]	<0.001 *
Weeks since COVID-19	*r* = −0.819 [−0.865; −0.753]	<0.001 *	*r* = 0.701 [0.593; 0.782]	<0.001 *
PCFS scale	*r* = 0.811 [0.744; 0.866]	<0.001 *	*r* = −0.466 [−0.640; −0.297]	<0.001 *
PAPs	*r* = 0.708 [0.623; 0.778]	<0.001 *	*r* = −0.781 [−0.850; −0.699]	<0.001 *
FAC	*r* = −0.640 [−0.736; −0.523]	<0.001 *	*r* = 0.647 [0.524; 0.742]	<0.001 *
LVEF	*r* = −0.640 [−0.557; −0.295]	<0.001 *	*r* = 0.617 [0.498; 0.721]	<0.001 *
E/e’	*r* = 0.435 [0.281; 0.567]	<0.001 *	*r* = −0.452 [−0.606; −0.289]	<0.001 *
HADS-D	*r* = 0.726 [0.630; 0.802]	<0.001 *	*r* = −0.652 [−0.767; −0.505]	<0.001 *
HADS-A	*r* = 0.440 [0.290; 0.577]	<0.001 *	*r* = −0.702 [−0.783; −0.583]	<0.001 *

Legend: QoL VAS—quality of life visual analog scale; CRP—C reactive protein; PCFS—post COVID functional status; PAPs—systolic pressure in the pulmonary artery; FAC—fractional aria change; LVEF—left ventricular ejection fraction; Doppler; E/e′—early mitral inflow diastolic velocity E to average e′ velocity (E/e′) in pulsed tissue Doppler; HADS-D and HADS-A—Hospital Anxiety and Depression subscale for Depression and Anxiety; Spearman correlation; r-sample correlation coefficient; CI—confidence interval; a *p* value ˂ 0.05 was considered statistically significant; * a significance threshold value of 0.05 was reached.

**Table 3:** Test results concerning the assessment moment.

	Evaluated during the First 6 Weeks 82 Subjects (57.34%) 48 Inpatients/34 Outpatients	Evaluated during Weeks 7–11 61 Subjects (42.65%) 16 Inpatients/45 Outpatients	*p* Value
Number of persisting symptoms	5 [4–6]	3 [2–3]	<0.001 ^a^*
PCFS scale: level: 1	15 (18.29%)	43 (70.49%)	
2	35 (42.68%)	18 (29.5%)	<0.001 ^b^*
3	32 (39.02%)	0 (0%)	
QL-5D-5L: mobility	-	-	-
self-care	-	-	-
activity: L2	36 (43.9%)	53 (86.88%)	
L3	32 (39.02%)	8 (13.11%)	<0.001 ^b^*
L4	14 (17.07%)	0 (0%)	
pain/discomfort: L2	54 (65.85%)	61 (100%)	<0.001 ^b^*
L3	28 (34.14%)	0 (0%)	
anxiety-depression: L2	16 (19.51%)	41 (67.21%)	
L3	36(43.09%)	18 (29.5%)	<0.001 ^b^*
L4	30 (36.58%)	2 (3.27%)	
VAS score	60 [48.75–66.25]	75 [66.5–78]	<0.001 ^a^*
HADS D	11 [10–18]	8 [6–10]	<0.001 ^a^*
HADS A	12 [8.75–17]	8 [6–10]	<0.001 ^a^*

Legend: PCFS—post COVID functional status; L—level; VAS—visual analog scale; HADS-D and A—Hospital Anxiety and Depression subscale; D—depression; A—anxiety; []—interquartile range; ^a^ Mann-Whitney U test; ^b^ Fisher’s exact test; *p* value ˂ 0.05 was considered statistically significant; * significance threshold value of 0.05 was reached.

## Data Availability

The data are available at http://dx.doi.org/10.17632/z73f3dhs6m.1, 15 June 2021.
